# Bacteriophage T4 Nanoparticles as Materials in Sensor Applications: Variables That Influence Their Organization and Assembly on Surfaces

**DOI:** 10.3390/s90806298

**Published:** 2009-08-12

**Authors:** Marie J. Archer, Jinny L. Liu

**Affiliations:** U.S. Naval Research Laboratory/4555 Overlook Ave., SW, Center for Biomolecular Science and Engineering, Washington, D.C. 20375, USA; E-Mail: jinny.liu@nrl.navy.mil

**Keywords:** bacteriophage T4, sensors, atomic force microscopy

## Abstract

Bacteriophage T4 nanoparticles possess characteristics that make them ideal candidates as materials for sensors, particularly as sensor probes. Their surface can be modified, either through genetic engineering or direct chemical conjugation to display functional moieties such as antibodies or other proteins to recognize a specific target. However, in order for T4 nanoparticles to be utilized as a sensor probe, it is necessary to understand and control the variables that determine their assembly and organization on a surface. The aim of this work is to discuss some of variables that we have identified as influencing the behavior of T4 nanoparticles on surfaces. The effect of pH, ionic strength, substrate characteristics, nanoparticle concentration and charge was addressed qualitatively using atomic force microscopy (AFM).

## 1. Introduction

Recently, the use of plant and bacterial viruses (bacteriophages) as constitutive elements of diagnostic systems and devices has gained interest due to their wide range of sizes and shapes, their robustness and, the possibility to tailor their surface with functional moieties. This last feature can be attained through genetic engineering (display systems) or direct chemical conjugation on wild type or engineered viruses [1–7]. In sensor applications, viruses have been used as biorecognition elements in surface plasmon resonance (SPR) [8–9], quartz crystal microbalance (QCM) [10], magnetoeleastic [11–13], and CMOS [14] sensors, among others. The selectivity is provided either by the viruses inherent recognition properties against certain bacteria or, by engineering their surface to express moieties for specific recognition against a target. Detection of *B. Anthracis* spores [12], *Salmonella typhimurium* [11], *E. coli* [14–16], *Staphylococus aureus* [9] and β-galactosidase from *E. coli* [8,10] has been demonstrated.

**Figure 1.**
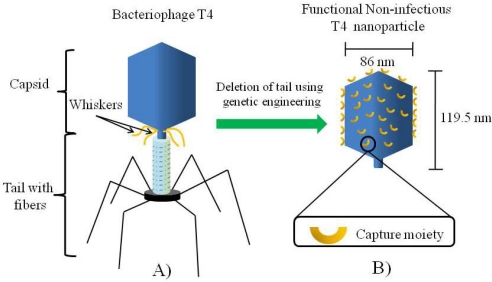
Schematic representation of A) bacteriophage T4 with all its native structures (capsid, tail, whiskers and tail fibers) and B) the non-infectious functional T4 nanoparticle decorated with a capture moiety resulting from genetic engineering of the wild type bacteriophage T4.

Among the broad range of plant and bacterial viruses that have been investigated, the interest in the use phages and particularly bacteriophage T4 as a nano-material, has recently increased, due to its flexible, unrestricted display system [3,17–18]. In comparison, plant viruses such as the Cowpea Mosaic Virus (CPMV), are not suitable for use as display systems. Site directed mutagenesis can be done to express small peptides on a specific number of sites onto which a moiety can be conjugated. This limits their use as templates for chemical conjugation and even if used as biorecognition elements in sensors, the restricted number of moieties would significantly reduce the sensitivity. In other phage display systems such as the M13, phage lambda and bacteriophage T3, among others, the display of long peptides is restricted since it affects the assembly of the phage and hence its biological properties [3]. The basic makeup of bacteriophage T4 is a capsid which contains densely packed DNA and a tail with specialized structures (fibers) used to anchor onto the cell surface of *E.coli* and inject the DNA during infection. For material and sensor applications, non-infectious T4 nanoparticles, consisting only of the capsid, or the capsid and the whiskers, can be synthesized by deletion of the tail through genetic engineering. This deletion can be accompanied with surface engineering to express capture moieties specific for a particular target leading to functional T4 nanoparticles for use as biorecognition elements in sensor devices. Figure 1 shows a schematic representation of this concept.

**Figure 2.**
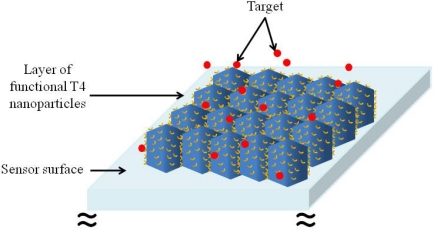
Schematic representation of a sensor surface which utilizes functional T4 nanoparticles as biorecognition elements. The detection of the target could be done through optical or electrical transduction.

Although, the use of T4 nanoparticles in biotechnology has been proposed and demonstrated for applications such as diagnostic imaging, vaccine development and detection of targets in liquid phase [3,4,17,19], only the whole bacteriophage T4 (capsid and tail structure–Figure 1A) has been demonstrated as constituent of a CMOS based sensor for detection of *E. coli* [14]. For this purpose, the bacteriophage T4 was positioned on a dielectric layer, presumably with the tail structure facing outwards, in order to anchor onto the membrane of *E. coli*. To the best of our knowledge, there is no previous report on any attempt to use T4 nanoparticles as probes on any type of detection system. For T4 nanoparticles to be used as biorecognition elements in sensors it is necessary to control their assembly on a surface. In order to take advantage of the whole surface area of the capsid and hence, increase the sensitivity of the sensor, it would be highly desirable to have a one dimensional layer of functional T4 nanoparticles arranged in close proximity to each other, in a similar fashion as a mosaic. This concept is schematically shown in Figure 2.

The assembly of viral particles on surfaces has been investigated in detail for the well characterized Cowpea Mosaic Virus (CPMV) and the results obtained demonstrate that assembly is dominated by a different set of factors than those observed in small molecule epitaxial systems [20]. Although these results are of great value, they may not have a direct correlation with T4 nanoparticles, given the many differences in their shape, size and protein distribution. Based on these characteristics, and the differences in the overall surface charge profile, it is likely that the behavior of T4 nanoparticles differ significantly from that of CPMV. Up to the present time there has been no assessment, experimental or theoretical, of the variables involved in the assembly and organization of T4 capsids on surfaces.

In considering the results presented here it is important to keep in mind that, from our perspective, for sensor development applications, ideal conditions are those that facilitate the distribution of the T4 nanoparticles without aggregation and with maximum surface coverage. The aim of the work presented here is to discuss some of the variables that we have identified as influencing the behavior of T4 nanoparticles on surfaces. For this purpose, the effect of pH, ionic strength, substrate characteristics, nanoparticle concentration and charge was addressed qualitatively using atomic force microscopy (AFM). The questions that rise are, first, which if any of the variables under study dominates over the assembly and organization of the T4 nanoparticles on a surface, and second, are these only variables involved in the organization and self-assembly of the T4 nanoparticles. The results presented in this work will provide insight into the variables that are likely to affect the behavior of T4 nanoparticles as a material, although it will not give a definite answer these questions.

## 2. Results and Discussion

The effect of buffer pH, ionic strength, T4 nanoparticle concentration and substrate characteristics on the assembly and organization of T4 nanoparticles was evaluated using atomic force microscopy (AFM). During these experiments we determined that media with high ionic strength (above or 100 mM), particularly the presence of excessive sulfate and sodium chloride, which result from the purification process, produced aggregation of the heads when used for permanent storage. Dilution of the stock solutions in salt free buffers followed by sonication did not alleviate the problem. However, we found that the use of 10 mM Tris-HCl was suitable in minimizing aggregation and for this purpose the rest of the characterization was performed using Tris-HCl based buffers.

### 2.1. Effect of the Media pH and Surface Charge on T4 Nanoparticle Aggregation and Surface Coverage

**Figure 3.**
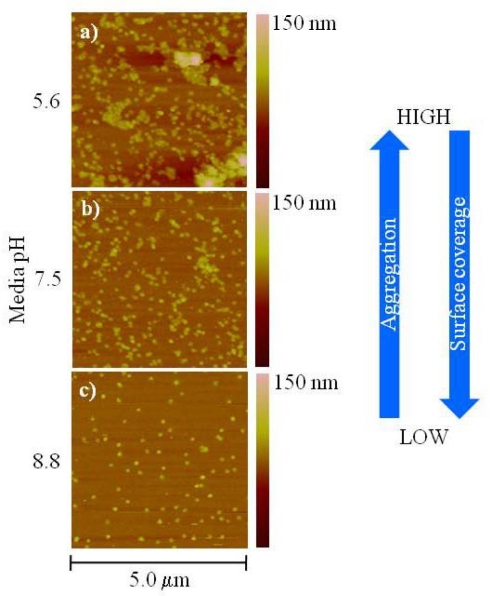
Atomic force microscopy (AFM) topographical images of T4 nanoparticles deposited on aminosilanized glass. In each case a 1:50 dilution from the stock solution was made in 10 mM Tris-HCl at pH a) 5.6, b) 7.5 and c) 8.8.

The selection of an aminosilanized substrate and the conditions for their preparation was based on the notion of providing a surface that would remain with a fixed charge within the range of pH tested. This would enable us to investigate the influence of the pH on the T4 nanoparticle by itself. It is worth mentioning that, at the present time the protein structure of the capsid has not been resolved and therefore it is difficult to determine the number of primary amines and carboxyl groups present. Also, the isoelectric point (pKa) of the T4 capsid as a single unit has not been determined and its charge at a given pH cannot be predicted. However, previous studies have determined the pKa value for the major head protein components and other structures and the results demonstrate a range between 6.2 and 4.8 for the capsid proteins that were investigated [21,22]. Based on these results and the fact that the whole bacteriophage (capsid, tail and fibers) has an isoelectric point close to 4, we would expect that the capsid acquire a negative charge above this pH, however it is not possible to determine the exact distribution of positive and negative charges on the capsid. Figure 3 shows atomic force microscopy (AFM) topographical images of T4 nanoparticles deposited on aminosilanized glass using 10 mM Tris-HCl at different pH.

From Figure 3, it can be seen that aggregation and surface coverage increases with a decrease in the buffer pH. We believe that the aggregation at low pH is due to a change in the overall charge balance on the capsid as a result of protonation and/or deprotonation of the various functional groups, mainly primary amines and carboxyl, which facilitates interactions among the nanoparticles. As mentioned before, from these results, the exact distribution of the charges and/or the orientation of the capsids with respect to each other in the aggregate cannot be determined. This is a complex scenario and interactions other than pure electrostatic ones might be influencing the capsid aggregation. PH dependent aggregation has been observed in other viruses, interestingly aggregates formed at low pH as well in structurally similar bacteriophages (bacteriophage T2) [23]. To test whether this effect was reversible we dialyzed a T4 nanoparticle solution in 10 mM Tris-HCl at pH 5.6 against the corresponding buffer at pH 8.8 for 12 hrs at room temperature. We found that the degree of aggregation was reduced which is consistent with the results observed previously [23].

To understand the dependence of the surface coverage with the pH it is necessary to consider the charge of the primary amines on the aminosilanized substrate as well as on the amino acid residues on the capsid at a given pH. The pKa of the primary amines is ∼9 and therefore they will likely bear a positive charge at pH below 9. On the other hand, the pKa of the carboxyl groups is ∼2 and therefore they will likely bear a negative charge at pH above 2. All three conditions presented in Figure 3 favor a positive charge on the aminosilanized substrate (pH below 9) suggesting that the variation on the surface coverage is driven by a change in the electrostatic interactions between the T4 nanoparticles and the surface. For instance, at pH 5.6 the increase in the surface coverage may indicate that the T4 nanoparticle aggregates behave as negatively charged entities favoring their electrostatic attraction with the strongly positively charged amine surface. This is in accordance with the expectation of the pKa value based on the capsid proteins component and the whole bacteriophage mentioned previously (∼4). Conversely, at pH 8.8 the amine surface is only slightly positively charged and the electrostatic interactions between the T4 nanoparticles and the surface become weaker. This same principle can be used to explain the change in T4 nanoparticle aggregation with pH. At pH 5.6 and 7.5, where the carboxyl groups are negatively charged and the amine groups are positively charged the agglomeration might be driven by electrostatic attraction between the carboxyl and amines among neighboring nanoparticles. As the pH is increased to 8.8 amines on the T4 nanoparticle are only slightly charged and the interactions between amine and carboxyl groups are minimized hence reducing agglomeration.

**Figure 4.**
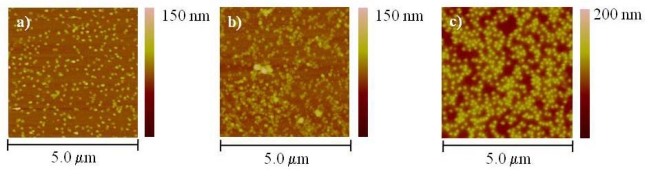
Atomic force microscopy (AFM) topographical images of T4 nanoparticles deposited at a 1:10 dilution in 10 mM Tris-HCl at pH 8.8 on a) bare (clean) glass, b) aminosilanized glass and c) freshly cleaved mica.

In order to further investigate how the surface charge influences the surface coverage we performed experiments using bare (clean) glass, aminosilanized glass and freshly cleaved mica. We used a slightly higher concentration of the T4 nanoparticles (1:10 dilution) in 10 mM Tris-HCl buffer at pH 8.8 in order to determine if there was any further enhancement in the surface coverage. The results are presented in Figures 4 a–c, respectively.

Comparison among the two glass substrates shows a higher surface coverage for the aminosilanized glass than for bare (clean) substrate. As mentioned before, the pKa of the primary amines is ∼9, and even though there is less positive charge at pH 8.8 there is still some adsorption of particles on the surface. On the other hand, the pKa of the hydroxyl groups of the bare (clean) glass is 4 and therefore they will likely bear a negative charge at pH 8.8. Based on the pH dependence experiments presented in the previous section, we expected that at this pH the T4 nanoparticles were likely to behave as negatively charged entities and therefore electrostatic repulsion between the glass surface and the nanoparticles will occur. It is worth to emphasize that the fact that there is some surface coverage, even on the negatively charged surface, suggests that the adsorption of the T4 nanoparticles might not be purely electrostatic but rather a combination of various types of interactions. As with the bare (clean) glass, mica will likely be negatively charged at pH 8.8 since the pKa of the siloxy groups is 6.8. Interestingly, comparison between either of the glass substrates and mica shows that the later has a significantly better coverage than glass suggesting that in mica the electrostatic repulsion is overcome by other mechanisms, mainly by the presence of counterions. It is well known that counterions present in low ionic strength buffer, such as the one used in these experiments, facilitate the interactions between the mica surface and other proteins and macromolecules [24]. As a matter of fact, AFM imaging of negatively charged macromolecules (such as DNA) can be successfully done using muscovite mica since screening of the negatively charged backbone by counterions allows its adsorption on the substrate at neutral pH. We believe that a similar mechanism is taking place with the T4 nanoparticles.

The change in the adsorptive properties of viruses as a result in the change in pH on modified surfaces (among them with a primary amine) has been systematically studied and has also been explained as a change in the ionization state of the surface and the residues of the virus capsid [25].

### 2.2. Effect of T4 Nanoparticle Concentration on Surface Coverage

**Figure 5.**
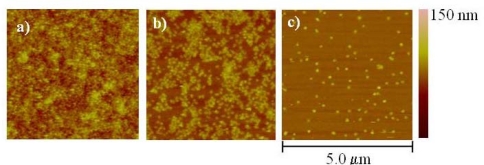
Atomic force microscopy (AFM) topographical images of T4 nanoparticles deposited on aminosilanized glass with a) no dilution, b) 1:1 dilution in 10 mM Tris-HCl at pH 8.8 and c) 1:50 dilution in 10 mM Tris-HCl at pH 8.8.

The effect of T4 nanoparticle concentration on the surface coverage was further investigated using aminosilanized substrates. The objective was to determine the effect of an increase in concentration on the surface coverage. Figure 5a–c show the results obtained when the concentration of T4 nanoparticles was varied from no dilution to a 1:50 dilution in 10 mM Tris-HCl at pH 8.8. This buffer was selected based on our previous experiments in order to minimize the T4 nanoparticle aggregation. Mild sonication for 5–10 seconds prior to the deposition on the surface was used in all the conditions to aid in the monodispersion of the nanoparticles.

Although the surface coverage increases at high concentration (Figure 5a) there is a lack of uniformity and the surface is characterized by alternating regions of void space and densely packed particles. We believe that the “disorganized” arrangement of the particles on the surface might be produced by non-uniform drying of the layer and is driven by similar inter-particle forces that rise upon solvent evaporation during the formation of films such as lateral capillary forces, flotation and convection forces. The drying process of water during the deposition process is related to the lateral transport and distribution of species across the substrate [26,27]. In a systematic study of lateral drying of colloidal particle films, Salamanca and co-workers demonstrated that as water evaporates, particles tend to form a closed packed region in which a capillary pressure is generated within the water-filled inter-particle void space. They suggest that uniform drying can be attained by maximizing the capillary force achievable thin films of large particle (>200 nm) suspensions at low evaporation rates [27]. To account for the possible effect of the evaporation rate on the uniformity of the layer, we performed the deposition within a humid environment for up to 72 hours to reduce the evaporation rate and allow enough time for the particles to distribute over the surface. However, we were not able to overcome the disorganized and non-uniform patterns observed in Figure 5a,b. These effects were ameliorated only when the concentration of the T4 nanoparticles was reduced (Figure 5c), suggesting that high concentrations favor interaction among neighboring nanoparticles that appear as agglomerates on the surface. It is also interesting to notice the extent of surface coverage at a large concentration even considering that, under these conditions (pH 8.8), the surface bears a less positive charge and, as explained before electrostatic interactions with the particles are minimized. At the moment, based solely on these results and without any further information on the specific structure of the capsid, we cannot explain the aggregation and the increased surface coverage at high concentration. However, given the possibility that the capsid properties could be affecting the interaction with neighboring nanoparticles at high concentrations, we wanted to test if by changing the characteristics of the capsid this aggregation could be reduced.

This was investigated by incorporating a PEG moiety onto the capsid proteins which has a twofold purpose: it modifies the capsid surface charge and increases its hydrophilicity. The results are presented and discussed in the following section.

### 2.3. Effect of T4 Nanoparticle Capsid Properties on the Surface Assembly

**Figure 6.**
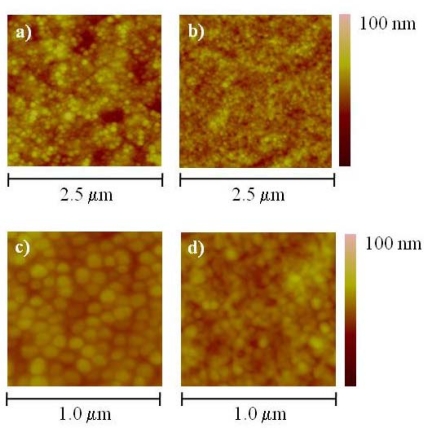
Atomic force microscopy (AFM) topographical images of poly(ethylene glycol) (PEG) derivatized (a and c) and a reference unmodified T4 nanoparticles on mica (b and d). The same nanoparticles concentration was used in both conditions.

Aggregation of the T4 nanoparticles at high concentrations represents an important issue for sensor development since it affects the control over their organization and assembly as a one dimensional layer. Evidently, based on the results presented here and the published literature, the characteristics of the capsid, mainly its charge, influenced by its protein composition, is one of the determinant factors on the aggregation and surface coverage. In order to evaluate the feasibility to control aggregation by modifying the capsid surface charge, we derivatized the surface with poly(ethylene glycol) (PEG) and deposited the modified T4 nanoparticles in the manner previously described, onto freshly cleaved mica. Figure 6 shows a comparison of the AFM topographical images between the PEG-derivatized T4 nanoparticles (Figure 6a and c) and the corresponding unmodified ones (Figure 6b,d).

Individual nanoparticles with delimiting borders can be identified in Figures 6a and 6c which correspond to the PEG derivatized T4 nanoparticles. It is interesting to notice the striking difference in size and shape, particularly evident in the low scale scanning (Figure 6a,d), between the PEG derivatized and the unmodified nanoparticles. From Figure 6a and 6c, it is evident that the PEG modification changes the interaction among particles and reduces their aggregation. Although we cannot explain the apparent difference in size and shape between the PEG derivatized and the unmodified nanoparticles solely based on these experiments, we speculate that the layer formed with the PEG derivatized nanoparticles could have a higher level of hydration which reduces the evaporation rate, helps preserve their size and shape even after drying the surface. We also believe that a change in the surface charge of the capsid after derivatization likely affects the electrostatic interactions between the capsids by reducing attractive forces. Additionally, the layer of hydration provided by the PEG moiety might be acting as a lubricant between the particles facilitating their distribution over the surface. The unmodified particles will be more susceptible to the effects of dehydration which leads to shrinking or collapse of the capsid. Also, the proximity to each other due to the high concentration of the sample might accentuate their electrostatic interactions facilitating the aggregation. Collapsed, aggregated capsids will certainly exhibit a different size range and shape. Just recently, the effect of dehydration on the structural integrity of viral capsids was characterized theoretically and experimentally by Carrasco and co-workers [28]. Collapse of viral capsids seems to be largely influenced by the forces exerted by water during the drying process. Of particular relevance is the formation of water menisci inside and outside the capsid that generate strong enough capillary forces to deform or even break the capsid. The presence of a suitable layer of hydration around the capsid, such as the one provided by the PEG moiety, seems to be critical factor to preserve the capsid integrity and to control their organization and arrangement on the surface.

The change in the charge of the PEG derivatized nanoparticles was further confirmed through an observable difference in the electrophoretic mobility between the PEG derivatized and the unmodified nanoparticles in an ethidium bromide stained agarose gel (data not shown). The PEG derivatized nanoparticles migrated further towards the anode than the unmodified ones suggesting an increase in their overall negative charge as a result of the reaction of the NHS-ester of the PEG with the primary amines on the capsid. An additional variable that has not been discussed is the possibility of the nanoparticles laying in a preferential orientation on the surface, either horizontally or vertically. Analysis of the height measurement from the topographical images on samples deposited at low concentration indicates that the nanoparticles are lying horizontally. The average length of single particles was between 100 to 120 nm while the height was ∼60 nm. The difference in height with respect to the expected theoretical value (∼90 nm) is likely the result of dehydration and partial collapsing of the nanoparticles as previously explained. An additional parameter that has to be considered when analyzing these results is the interaction of the AFM probe with the nanoparticles. This interaction will vary depending on the hydrophilicity of the surface. However, it is difficult to attribute any particular aspect of the results presented here to the nanoparticle-tip interactions without further and more detailed experimentation.

From the results obtained in this study, we believe that the variables that play a significant role in the assembly of T4 nanoparticles are the capsid charge, which changes with the media pH, and the level of hydration. The protein composition of the capsid determines its charge distribution which changes with the media pH. Therefore modification of the capsid (by chemical conjugation) could be used to tailor effects such as aggregation and surface coverage. On a solid surface, changes in the media pH will modify the interactions not only between the neighboring capsids, but also between the capsids and the surface. Manipulation of the capsid charge through change in the residue chemistry and/or media pH could be used as handles to tailor the conditions for optimal surface coverage and reduced aggregation. The level of hydration also influences the interaction between neighboring capsids, and therefore their assembly, by modifying the forces exerted during the drying process. Additionally, hydration determines the integrity and shape of the capsid. It is worth mentioning that the effect of hydration was not considered at the beginning of this study. However, the experiments performed with the PEG moiety, which were initially intended to modify the surface charge, provided us with this interesting finding which we believe is critical for sensor development.

## 3. Experimental Section

*Reagents and solutions.* All solutions were prepared with Milli-Q water. Tris-HCl buffer (10 mM) at pH 8.8 was purchased from Ambion (Applied Biosystems/Ambion, Austin, TX, USA). 1 M Tris-HCl buffer at pH 7.5 was purchased from Sigma Aldrich (St. Louis, MO, USA) and diluted to 10 mM with Milli-Q water. Trizma hydrochloride was purchased from Sigma Aldrich to prepare 10 mM Tris-HCl and the pH adjusted to 5.6 with 0.1 M sodium hydroxide. Sulfuric Acid, hydrogen peroxide (30% wt), 3-(aminopropyl)triethoxysilane (APTES), 100% absolute ethanol and dimethylsulfoxide (DMSO) were purchased from Sigma Aldrich and used as received. Phosphate-buffered saline (0.01 M) was purchased from Sigma Aldrich and prepared by dissolving one foil pouch in 1 liter of Milli-Q water (0.138 M NaCl, 0.0027 M KCl, pH 7.4). For poly(ethylene glycol) derivatization, Methyl-PEO_4_-NHS ester reagent (arm spacing of 16.4 Å) was purchased from Thermo Fisher Scientific (Rockford, IL, USA) and dissolved in 1.1 ml of anhydrous dimethlysulfoxide (DMSO).

*Preparation of the T4 non-infectious capsid.* T4 K10 (38^−^ 51^−^ *denA*^−^ *den*B^−^), a kind gift from Dr. Lindsay Black at University of Maryland at Baltimore Medical School, was first propagated in the suppressor *E. coli* host strain, CR63, to obtain the infectious phage [29]. The infectious phage were then used to produce non-infectious capsids in the non-suppressor host *E. coli* strain, Rosetta, modified from a previous procedure [30]. In brief, Rosetta grown in M9S (OD_600_ = 0.5) were infected with K10 phage at moi = 3 for 2 hrs at 37 °C. Cells were spun down and resuspended in 10 mM Tris-HCl (pH7.5) supplementing with 2 mM MgCl_2_ and 1 mM CaCl_2_. CHCl_3_ (1/20 of total volume), DNaseI (20 μg/mL) and RNase I (50 μg/mL) were then added to the cell suspension and shaked at 37 °C for 1 hr. The cell debris was removed after spinning at 8,000 rpm for 30 min. The cell lysate was concentrated through micorcon YM-100 membrane according to the manufacturer’s procedure (Millipore Corp., Billerica, MA, USA). The membrane was then washed with 10 mM Tri-HCl and 2 mM MgCl_2_ for six times. The non-infectious capsids retained on the membrane were resuspended in 10 mM Tri-HCl buffer for AFM imaging.

*Derivatization of T4 nanoparticles with poly(ethylene glycol)*. For derivatization with poly(ethylene glycol), 10 μl of the stock solution were re-suspended in 480 μl of 1X PBS prepared as described before. Ten μl of Methyl-PEO_4_-NHS ester reagent was then added and the T4 nanoparticles were incubated at room temperature for 3 hours on a dry block thermomixer (Eppendorf, West Bury, NY, USA). After incubation, desalting and removal of excess pegylation reagent was performed using a micorcon YM-100 membrane according to the manufacturer’s procedure (Millipore Corp.). The T4 nanoparticles were resuspended in 10 mM Tris-HCl with 2 mM MgCl_2_ at pH 7.5.

*Preparation of glass and mica substrates.* Circular borosilicate glass cover slips (12 mm in diameter, 0.13–0.17 thick) were purchased from SPI Supplies (West Chester, PA, USA). Prior to use, the cover slips were cleaned in a 3:1 v/v solution of sulfuric acid (H_2_SO_4_): hydrogen peroxide (H_2_O_2_) 30% wt (piranha etch) at 85 °C for 15 minutes. The cover slips were then rinsed thoroughly with DI water, dried with nitrogen and stored in ethanol until their use. Aminosilanization was performed immediately after cleaning of by immersion in a 10% solution of APTES prepared in 95% ethanol for 1 hr, at room temperature with gentle stirring. After aminosilanization, the cover slips were rinsed twice with 95% ethanol, twice with Milli-Q water, dried with nitrogen and cured in a dry oven at 120 °C for 3 hrs. The aminosilanized cover slips were stored in air tight containers and used within 24 hours of preparation. Muscovite mica discs (12 mm diameter) were purchased from SPI supplies (West Chester, PA, USA) and cleaved prior to deposition of the T4 nanoparticles.

*Sample preparation.* Fresh dilutions of T4 nanoparticles were prepared immediately before deposition on glass or mica substrates at required concentrations from a stock solution of 5 × 10^12^ particles/ml. For the pH dependence experiments a 1:50 dilution of the stock solution was made in 10 mM Tris-HCl at either pH 5.6, 7.5 or 8.8. For the nanoparticles concentration dependence experiments, undiluted samples were used directly from the stock and serial dilutions of 1:10 through 1:50 were done using 10 mM Tris-HCl at pH 8.8. Five μl of the prepared T4 nanoparticles were deposited on aminosilanized glass, clean glass or freshly cleaved mica and kept in a humid environment at room temperature for 12 hours. After adsorption of the T4 nanoparticles onto the surface, the substrates were allowed to dry at room temperature and then carefully rinsed with Milli-Q water.

*AFM imaging and data analysis.* Imaging was performed using a Multimode Scanning Probe Microscope (Veeco Instruments, Plainview, NY, USA) in air under tapping mode using a commercial silicon cantilever (NanoScience Instruments, Phoenix, AZ, USA), 125 μm long, with an apex curvature radius of 5–6 nm, a resonant frequency of 300 kHz and spring constant of 40 N/m. The scanning rate was 0.5 Hz, at 0° angle. Image processing was performed using Research NanoScope II software version 7.20. All images were filtered using the flattening built-in tool from NanoScope II software. Length and height values of the nanoparticles were obtained by utilizing the built-in tool for cross sectional analysis. Height and length values of at least twenty individual T4 nanoparticles were obtained from the section data and averaged.

## 4. Conclusions

In this work we have investigated some of the variables that influence the organization and assembly of T4 nanoparticles on surfaces using Atomic Force Microcopy (AFM). Knowledge of these variables is relevant for their use in sensor applications. Our findings suggest that, the surface of the T4 nanoparticle capsid plays a significant role in determining their organization however we do not believe that this is the only variable involved. The best conditions under which an organized assembly of T4 nanoparticles seems to be favored are using freshly cleaved mica as a substrate, in a low ionic strength buffer at pH 8.8 (10 mM Tris-HCl) with T4 nanoparticles modified with a Polyethylene(glycol) (PEG) moiety. While the pH of the media and the characteristics of the surface play an important role, we found that the aggregation of the T4 nanoparticles was significantly reduced upon modification of the capsid surface with a PEG moiety. We believe that the organization and assembly of T4 nanoparticles could be controlled to form a one dimensional layer by modifying the nanoparticles interaction through a change in the capsid charge along with the characteristics of the substrate onto which is deposited. Our observations that a hydrated environment might be aiding in preserving the integrity of the capsid leads us to believe that AFM analysis in liquid is required to obtain more conclusive information about these and other variables.
